# Facility Attractiveness and Social Vulnerability Impacts on Spatial Accessibility to Opioid Treatment Programs in South Carolina

**DOI:** 10.3390/ijerph18084246

**Published:** 2021-04-16

**Authors:** Parisa Bozorgi, Jan M. Eberth, Jeannie P. Eidson, Dwayne E. Porter

**Affiliations:** 1 Department of Environmental Health Sciences, Arnold School of Public Health, University of South Carolina, Columbia, SC 29208, USA; porter@sc.edu; 2 South Carolina Department of Health and Environmental Control (SCDHEC), Columbia, SC 29201, USA; eidsonjp@dhec.sc.gov; 3 Rural and Minority Health Research Center, Arnold School of Public Health, University of South Carolina, Columbia, SC 29210, USA; jmeberth@mailbox.sc.edu; 4 Department of Epidemiology and Biostatistics, Arnold School of Public Health, University of South Carolina, Columbia, SC 29208, USA

**Keywords:** Geographic Information Systems (GIS), spatial accessibility, opioids, access to care, catchment area

## Abstract

Opioid dependence and opioid-related mortality have been increasing in recent years in the United States. Available and accessible treatments may result in a reduction of opioid-related mortality. This work describes the geographic variation of spatial accessibility to opioid treatment programs (OTPs) and identifies areas with poor access to care in South Carolina. The study develops a new index of access that builds on the two-step floating catchment area (2SFCA) method, and has three dimensions: a facility attractiveness index, defined by services rendered incorporated into the Huff Model; a facility catchment area, defined as a function of facility attractiveness to account for variable catchment size; and a Social Vulnerability Index (SVI) to account for nonspatial factors that mitigate or compound the impacts of spatial access to care. Results of the study indicate a significant variation in access to OTPs statewide. Spatial access to OTPs is low across the entire state except for in a limited number of metropolitan areas. The majority of the population with low access (85%) live in areas with a moderate-to-high levels of social vulnerability. This research provides more realistic estimates of access to care and aims to assist policymakers in better targeting disadvantaged areas for OTP program expansion and resource allocation.

## 1. Introduction

In the United States, drug-overdose deaths have more than tripled from 1999 to 2018. In 2018, opioid overdose was involved in almost 70% of these deaths [1]. In 2019, a total of 1131 drug-overdose deaths occurred in South Carolina, a 2.5% increase from 2018 with 77.4% involving an opioid. From 2018 to 2019, deaths involving all opioids, prescription opioids, and heroin increased by 7.4%, 7%, and 16%, respectively [2].

Three medications are currently approved by the Food and Drug Administration (FDA) to treat opioid dependence: methadone, buprenorphine, and naltrexone [3]. Due to the risk of abuse and overdose, methadone is only dispensed from tightly regulated opioid treatment programs (OTPs) licensed by the US Substance Abuse and Mental Health Services Administration (SAMHSA) [3].

Studies show that using these medications reduces the risk of overdose and opioid-related mortality [4,5]. Additionally, driving short distances for the treatment can decrease the rate of opioid-related mortality [6]. Despite the demonstrated effectiveness of the FDA-approved medications for the treatment of opioid-use disorder (OUD), studies have demonstrated low rates of treatment use [7]. In 2017, over 70 percent of people who needed treatment for OUD did not receive medication [8]. Of those in treatment, a minority (<30%) receive treatment with methadone or buprenorphine [9].

Previous studies have identified obstacles to receiving treatment, including poor accessibility and availability, treatment cost, lack of health insurance coverage, and lack of support services such as assistance with housing, child care, and transportation [10,11,12]. One study found that patients traveled an average of 49 miles to reach medication prescribers, and that those traveling a mean distance greater than 45 miles to prescribers were less likely to regularly receive medications [13]. Methadone is usually taken once daily under the supervision of a practitioner at OTP facilities [14], and daily travel over long distances can add the significant burden of transportation costs for most patients, especially for rural residents who need to travel a longer distance [15]. Longer travel distances have also been associated with shorter lengths of stay in outpatient methadone clinics and lower probabilities of treatment completion and aftercare utilization [13]. The distance to an OTP has also been associated with the number of missed doses in the first month of treatment. Specifically, patients who lived more than 10 miles from the OTP were more likely to miss doses compared to individuals who lived within 5 miles of the OTP [16].

While findings from these studies were critical in advancing our understanding of the importance of a geographic perspective on access to OTPs, inequality in spatial accessibility to OTPs in South Carolina has not been studied. Determining and evaluating geographic variations in spatial access to OTPs may help explain why some areas have higher rates of drug overdose or drug overdose deaths.

Access to care is a multidimensional concept influenced by both spatial and nonspatial factors that can be further categorized into potential and revealed accessibility. Revealed accessibility focuses on the actual use of health-care services, whereas potential accessibility considers the population as the potential users of health-care providers [17]. Spatial access to health care is primarily dependent on three factors: supply, demand, and travel costs between supply and demand. The two-step floating catchment area (2SFCA) method is based on the gravity model [17] that considers both supply and demand, as well as their interaction. First, it defines a catchment (service area) of a 30 min drive time around the facility and determines the population-to-provider ratio (PPR). The second step identifies a catchment around the demand location and searches for all facilities within the demand’s catchment area. Each facility found in a resident’s catchment area will have a corresponding PPR, calculated in step one. The spatial accessibility index is calculated by summing the PPR of all facilities within the demand catchment. The final 2SFCA score is computed in a two-step process (see Appendix A).

Despite the popularity of 2SFCA, the method has a drawback—it does not consider distance decay and assumes that all services within the catchment area are equally accessible. Additionally, it uses a fixed catchment size, which is more problematic for the comparison of urban and rural areas which may have very different commuting behaviors [18,19]. Modifications to the basic form of 2SFCA include: improvements of the method of calculating catchment size [20,21,22]; the inclusion of nonspatial factors and competitive effects among the facilities [23,24,25,26]; the incorporation of distance decay within catchments [27]; and the implementation of variable catchment sizes [20].

Spatial accessibility models have been widely used to measure access to different types of health-care facilities and services, including inpatient health care, mammography, cancer screening, and primary care [25,28,29,30]. However, geographic variation in accessibility to OTPs remains primarily unknown. This research develops a spatial access model that builds on the two-step floating catchment area (2SFCA) method and accounts for nonspatial factors and facility attractiveness. We expect that our model will reveal a more reasonable pattern than the traditional 2SFCA method. Specifically, this research examines spatial accessibility to OTPs in order to identify low and high spatial access areas in South Carolina.

The findings provide support for state and local governments to better allocate treatment resources where access to treatment is limited.

## 2. Materials and Methods

### 2.1. Overview

This study estimated facility attractiveness and used the Huff Model for quantifying the probability of a person’s preference for an OTP site, accounting for factors including distance to, and the attractiveness of, the OTP site. A key feature of the proposed model, besides measuring the attractiveness of the facility based on multiple attributes, was to integrate the Centers for Disease Control and Prevention (CDC) Social Vulnerability Index (SVI) to account for nonspatial factors. The facility catchment size was also determined as a function of facility attractiveness. We evaluated the relation between our model (i.e., the weighted 2SFCA (W2SFCA)) and the 2SFCA model using the Spearman correlation coefficient and the intraclass correlation coefficient (ICC). To assess whether high or low access scores cluster spatially, the hot-spot analysis with optimal distance band identified based on incremental spatial autocorrelation was used. Choropleth maps of the final accessibility indices highlight differences between the methods.

### 2.2. Study Area

A spatial accessibility model was calculated for block groups in South Carolina—A state located in the southeastern region of the US with a population of 5,148,714 over a 32,020 mi^2^ area—is characterized by rural and urban landscapes [31].

South Carolina has 46 counties and 3046 block groups. There are 21 OTPs statewide, with most of them clustered in urban areas and only 4 OTPs located in rural areas. From a demographic perspective, many of the counties (28 out of 46 counties) are classified as having highly vulnerable populations, based on their CDC SVI scores, which accounts for almost 30% of the state’s total population.

### 2.3. Data Sources

Information on OTPs was obtained from the publicly available Substance Abuse and Mental Health Services Administration (SAMHSA) data released in 2019. The data contained the location and services provided by facilities. The location of services was geocoded with the corresponding street addresses by our team. The list of service settings and treatment types for the centers is listed in Appendix B.

Population data were extracted at the block group level from the US Census Bureau’s Integrated Public Use Microdata Series (IPUMS), explicitly using the 2013–2017 American Community Survey. To represent population location more accurately, we calculated population-weighted block group centroids based on the Census block population. Distances between OTP service locations and demand locations were calculated based on the 2018 street network using Network Analyst of ArcGIS Pro (ESRI Inc., Redlands, CA, USA).

The Social Vulnerability Index (SVI) at the Census tract level was obtained from the 2017 Centers for Disease Control and Prevention (CDC) [32]. The SVI was created to identify socially vulnerable populations and rank US Census tracts based on the resident population’s demographics. It ranks four domains (Socioeconomic Status, Household Composition and Disability, Minority Status and Language, and Housing and Transportation), based on 2–5 demographic indicators, in addition to Overall Vulnerability, which aggregates all of the indicators into a single summary rank. We assumed that all the block groups within the Census tract have the same overall ranking as their Census tract.

### 2.4. Analysis

To address the limitations of previous accessibility models, our method focused on enhancing the provider catchment size and applying nonspatial factors in three steps.

In the first step, we defined facility catchment size as a function of facility attractiveness. To determine facility attractiveness, we developed a composite index of attractiveness based on factors including the type of opioid treatment provided, availability of counseling services, provision of detoxification, ancillary services provided, payment/insurance types accepted, and language services available (full list of services is included in Appendix B). A facility’s service was given more weight if the facility was located within an area where the majority of the population were vulnerable due to a lack of that service. For example, greater weight was allocated to the housing and transportation services provided by a facility if the site was located in an area where the majority of the population were classified in the highest vulnerability category for housing and transportation; otherwise, no weight was given to that service. Determination of the highest/lowest vulnerable population was based on CDC SVI scores (4 categories representing 0–25%, 25.01–50%, 50.01–75%, 75.01–100%). In this step, the facility attractiveness of a treatment facility *j* ($C_{j}$) was quantified as a sum of the weighted attributes mentioned earlier:

(1) $$C_{j}=\overset{n}{\underset{k=1}{\sum }}W_{k}X_{k}$$

where:

- $X_{k}$ is the *k*-th attributes assigned for treatment facility *j*;
- $W_{k}$ is the weight assigned to the attribute $X_{k}$.

A high score effectively increased the size of the population competing for access to the available services. Then, we used the Huff Model to estimate the most likely population accessing the facility. For each block group, we measured and/or created:

- a population-weighted centroid to represent the location of the demand population.
- the travel time between each block-group centroid and facility address, using the origin-destination (OD) cost-matrix function of ArcGIS Pro 2.3.
- an 80 min drive time catchment area around the demand location, calculated using the closest facility function of ArcGIS Pro 2.3.
- the Huff Model selection probability of a population location on each treatment facility within its catchment using Equation (2).

(2) $$Prob_{i}=\frac{C_{j}e^{{\frac{-d}{\beta }}^{2}}}{\sum_{s\in D_{0}}C_{s}e^{{\frac{-d}{\beta }}^{2}}}$$

where:

- $Prob_{i}$ is the Huff Model-based selection probability of population i at treatment facility *j*;
- $C_{j}$ is the attractiveness of treatment facility *j* calculated from the previous step;
- $d_{ij}$ is the shortest travel time from population *i* to treatment facility *j* and β is the distance impedance coefficient.

Calculation of the shortest travel time from the population centroid to the OTPs showed that an 80 min drive time ensured that each block group had access to at least one OTP within its catchment. The value of β was estimated using the Gaussian function (Equation (3)). A value of 0.01 was considered a threshold value when the distance decay function approaches 0 [33]. The Gaussian function was adopted as the distance decay function because it has proven superior to other functions in simulating the distance impedance effect [34].

(3) $$\begin{array}{cccccccc} f_{d}=e^{{\frac{-d}{\beta }}^{2}} & & & & & & & \beta =-\frac{d_{0}{}^{2}}{\ln0.01} \end{array}$$

In the second step, we defined the facility catchment size (*D*) as a function of the treatment facility attractiveness using the Gaussian function (Equation (4)). To differentiate the facility catchment size in urban and rural areas, we determined the facilities’ urban/rural status using the 2013 urban–rural classification from USDA’s Rural–Urban Commuting Area (RUCA) codes. RUCA codes classify US Census tracts using measures of population density, urbanization, and daily commutes. A facility within a metropolitan area (codes 1–3) was defined as urban; all other facilities were labeled as rural (codes 4–10). Among facilities located in rural areas, the facility catchment size (*D*) was based on a threshold of 60 min vs. 30 min drive time for facilities located in urban areas. Towards our goal of defining effective facility catchment sizes, we multiplied these numbers by the facility attractiveness formulated using the Gaussian function.

(4) $$\begin{array}{ccc} D=e^{-{\left(\frac{C_{j}-C_{m}}{C_{m}}\right)}^{2}}\;*\;30\;\min & & C_{j}\leq C_{m}\\ D=e^{-{\left(\frac{C_{j}-C_{m}}{C_{m}}\right)}^{2}}\;*\;60\;\min & & C_{j}\leq C_{m} \end{array}$$

where:

- *D* is the facility catchment size;
- $C_{m}$ is the maximum attractiveness score.

The facility catchment sizes ranged from 17.2–30 min in urban areas and 32.5–46.2 min in rural areas. Then, we calculated the provider-to-population ratio ($R_{j})$ using Equation (5).

(5) $$\underset{\;\;\left(i\in D_{0}\right)}{R_{j}=\frac{C_{j}}{\sum \;Prob_{ij}W_{ij}\;P_{i}}}\;W_{ij}=e^{{\frac{-d}{\beta }}^{2}}$$

where:

- $R_{j}$ is a provider-to-population ratio at treatment facility *j*;
- $P_{i}$ is a weighted population of block-group i;
- $D_{0}$ is a travel threshold;
- $W_{ij}$ is a travel impedance between i and j;

We weighted the numerator by the facility attractiveness because facilities offering more services are more attractive than others.

In the third step, we defined an 80 min drive time catchment area around the population-weighted block-group centroid. We then summed the ratios from all facility locations falling within this catchment area. However, to account for nonspatial factors, we considered the output of the CDC SVI index associated with each population location. A high SVI score effectively reduces a population catchment size due to the higher social vulnerability and associated service needs of the population. We expressed the accessibility score as:

(6) $$A_{i}=\sum_{\left(j\in D_{0}\right)}R_{j}W_{ij}\;Prob_{ij}\cdot SVI^{-1}$$

where:

- $A_{i}$ is the accessibility at population location *i*;

Areas with higher scores for $A_{i}$ are considered to have better spatial accessibility to OTPs.

Using the same datasets, our weighted 2SFCA (W2SFCA) model was compared with the original 2SFCA model. Choropleth maps were also generated using ArcGIS Pro, allowing for the visualization of our final accessibility index vs. the traditional 2SFCA method. Hot-spot analysis was also conducted to identify statistically significant clusters of high and low values based on the neighborhood of each block group.

## 3. Results

Measures of central tendency and dispersion among the two accessibility scores are shown in Table 1. We tested the association between the two methods with data measured continuously using the Spearman correlation coefficient method. A positive relationship was found with a coefficient of 0.73 and a p-value of 0.003 (Table 2).

**Figure 1:**
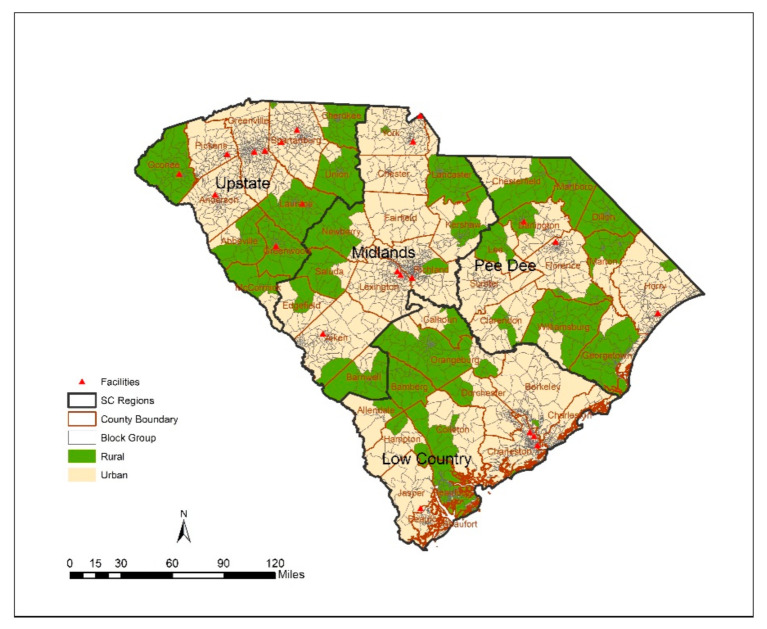
Study area and spatial distribution of opioid treatment program (OTP) facilities in South Carolina.

**Figure 2:**
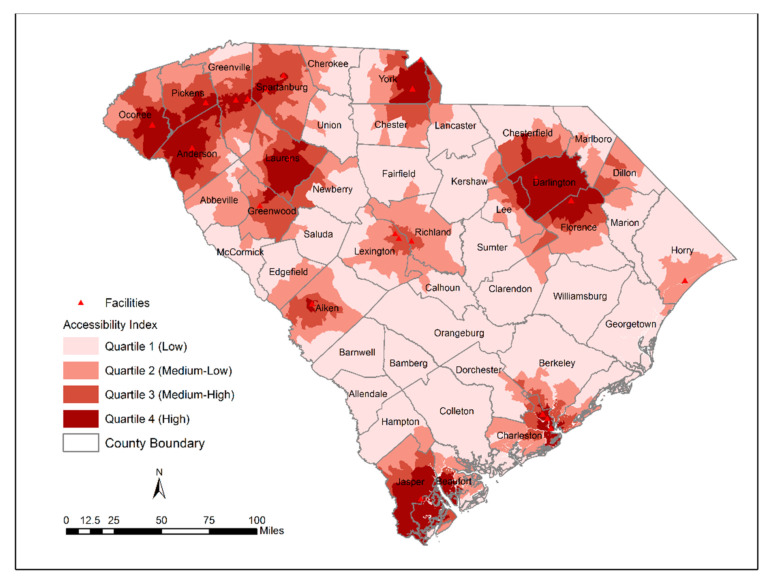
Distribution of W2SFCA access score.

**Figure 3:**
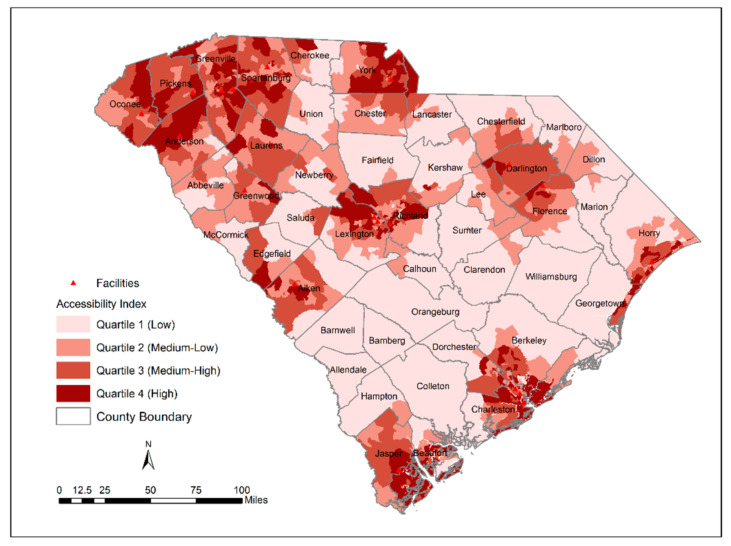
Distribution of W2SFCA access scores including Social Vulnerability Index (SVI).

The ICC was measured by a single-rating, 2-way random-effects model with two methods across 3046 block groups (Table 2). Although the obtained ICC value was 0.71 (indicating moderate reliability), a 95% confidence interval range between 0.2 and 0.8 means that there is a 95% chance that the true ICC value will land on any point between 0.2 and 0.8. Therefore, the level of reliability can be interpreted as poor to moderate. The geographic patterns of accessibility index computed by the W2SFCA (before and after including SVI) and the traditional 2SFCA model are shown in Figure 1, Figure 2 and Figure 3. The spatial distribution of accessibility by the W2SFCA (Figure 1) showed a relatively similar pattern to the traditional 2SFCA (Figure 3). However, the range of the accessibility scores by the W2SFCA was smaller than the range of the 2SFCA. For spatial comparison of the two methods, quantile classification groups with four classes were used.

According to the results obtained from the W2SFCA, shown in Figure 2, the spatial accessibility to OTPs is unevenly distributed. Areas with higher access were primarily located in the northern part of the state, with very few located in the south and north-east of the state. From the results of the accessibility analysis with the proposed method, approximately 21% of the state’s population lives in areas with low access; 23% live in areas identified as medium–low access; 26% live in areas identified as medium–high access; and 30% live in high-access areas. The majority of the population with low access (85%) live in areas with a moderate-to-high level of social vulnerability.

**Figure 4:**
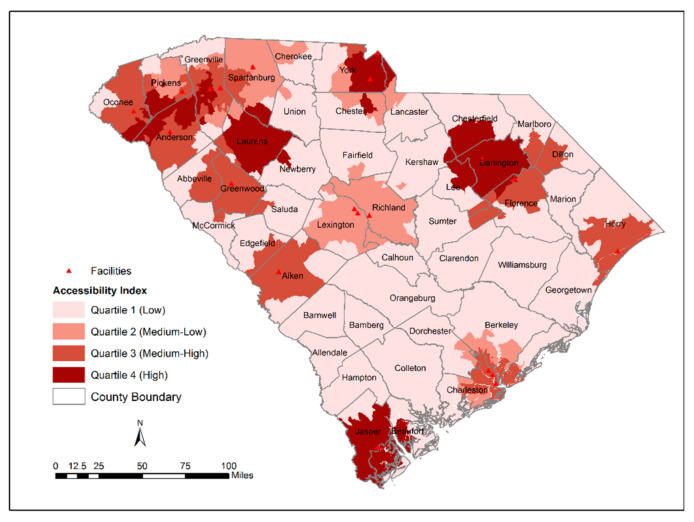
Distribution of 2SFCA access scores.

In comparison with the 2SFCA, as expected, the W2SFCA revealed more details of accessibility. For example, in the vicinity of OTPs located in Richland and Lexington counties, the accessibility is underestimated by the 2SFCA (Figure 4). The 2SFCA model detected all the block groups within these counties as areas with low accessibility while some of their block groups encompassed an OTP provider, and some were close to nearby OTP sites. This is because the 2SFCA method utilizes the fixed catchment size regardless of the attractiveness of the facilities. The SVI-weighted score revealed disparities in accessibility to OTPs relative to the socio-economic status of the population (Figure 3). As shown in Figure 3, some block groups adjacent to the OTP facility are identified as areas with low access within the Spartanburg city limits. People living in this area are ranked as a highly vulnerable population, and their socio-economic status can affect their access to OTPs. Some of these OTP facilities are among the facilities with the lowest attractiveness index, indicating that they either do not accept Medicaid/Medicare patients or do not provide additional services that can be beneficial for vulnerable populations.

**Figure 5:**
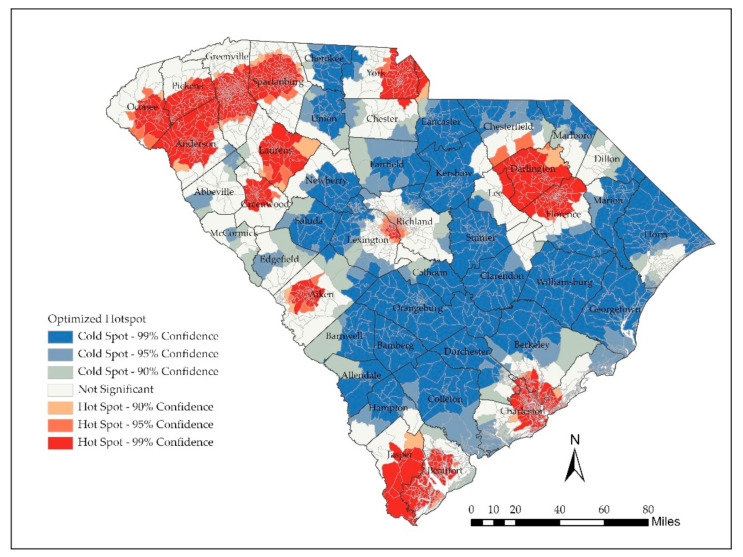
Hot-Spot Analysis of accessibility score (W2SFCA).

Results of the hot-spot analysis are shown in Figure 5. Cold spots with clusters of low accessibility were discovered in much of the Midlands, Pee Dee, and Lowcountry regions (with notable exceptions in Charleston, Beaufort, Darlington, and Florence Counties). Hot spots with clusters of high accessibility were clustered in the Upstate region, as well as Aiken County, the border of York and Lancaster Counties, and the counties listed above. Many of these hot spots were clustered near the metropolitan areas of the state or bordering states.

## 4. Discussion

The primary goals of this study were to explore the geographic variation of spatial accessibility to OTPs and to identify areas with poor accessibility in South Carolina. This paper outlines a new index of access that integrates facility attractiveness and socio-economic factors with the existing metrics. The facility attractiveness includes the services offered by the facility that help to measure each facility’s attractiveness to opioid users. Most previous studies use a distance-impedance coefficient β to create weights within the service catchment. These studies measure β by using the actual travel distance of patients who visited the treatment center. However, estimating β based on the empirical data is likely to be confounded with the existing distribution of facilities in a region instead of representing the patients’ inclination to travel to a facility. We defined facility catchment size as a function of facility attractiveness, formulated by the Gaussian function, to moderate its effect on spatial access measures for different impedance coefficients [23]. The SVI includes variables that help to identify populations that are more likely to have a lack of access to OTPs. The integration of these factors makes this approach more realistic and provides a better fit for modeling access to OTPs.

We compared our model with the 2SFCA method. We found that spatial accessibility is underestimated in some areas by using the 2SFCA method. This problem has been partially alleviated in the W2SFCA method by incorporating SVI and facility attractiveness into the model. We showed not only that being too far from a facility can result in decreased access to a facility, but also that sociodemographic factors and lack of accommodation at a facility (e.g., not accepting certain insurance plans) can present an obstacle to accessing care at that facility. Our findings have several public-health implications. They can be used for the identification of accessibility variations of OTPs throughout the state, and, possibly, for improving access to OTPs. Specifically, the scale of the analysis provides more granularity to uncover local areas of spatial homogeneity and heterogeneity for community-based interventions. Moreover, results of cluster analysis (e.g., clusters of low access) can be overlaid with the clustering of high rates of drug overdose to target interventions in areas where treatment programs are most needed. Our methodology is also deployable in other health-care facilities such as HIV care providers and mental-health services.

Despite the notable advantage of W2SFCA, several issues deserve attention when interpreting the results. Population locations used for this study are weighted block-group centroids. The developed method, however, has the potential to further articulate the population-selection behavior because the block-group population is not necessarily a proper indicator of opioid-treatment needs. This can be partially addressed in future development by incorporating the number of patients with a history of prescription opioid use or experience of opioid overdose. This study also assumes that all patients traveled by car and does not consider different modes of transportation, such as public transportation, as it is somewhat limited in the state. Moreover, it is possible to adjust the weights used for estimating the attractiveness score. Assigning different weights to the services might result in different accessibility scores. Different weighting scenarios can be implemented in future studies to assess sensitivity and robustness of the spatial accessibility score. Among treatments provided at OTP facilities, methadone currently needs to be taken under the supervision of a practitioner [14]; however, patients can take the treatment at home for maintenance purposes if they meet certain criteria. Policies to make take-home treatments more accessible should be considered to minimize the impact of geographic distance on treatment utilization. The impact of these policies on accessibility could be an important area of future spatial-accessibility research.

## 5. Conclusions

This study provides a new perspective for analyzing health-care accessibility, including both spatial and nonspatial factors to define the accessibility of OTPs in South Carolina. As stated in the introductory section, enhancing access to treatment can reduce the risk of overdose for individuals suffering from opioid-use disorders. The results of this study indicate a significant variation in levels of access to OTPs statewide. Rather than defining accessibility solely on the distance to OTP facilities, we considered the role of facility attractiveness and the social vulnerability of the potential demand populations. The traditional 2SFCA overestimates regional accessibility, and the W2SFCA can provide a more realistic evaluation. Based on this study, policymakers and public-health officials should consider optimizing the allocation of existing health-care resources or putting additional resources into low-accessibility areas.

## Figures and Tables

**Table 1:** Distribution of spatial accessibility scores.

Variable	Mean	Median	SD	IQR	Range
W2SFCA	0.00035	0.00036	0.00017	0.00028	0.00083
2SFCA	0.00024	0.00025	0.00020	0.00038	0.00091

**Table 2:** Analyses of agreement between the weighted two-step floating catchment area (W2SFCA) and the two-step floating catchment area (2SFCA).

		2SFCA
W2SFCA	Spearman’s Correlation ICC (95% CI)	0.73
		0.71 (0.21–0.86)

## Data Availability

Publicly available datasets were analyzed in this study. This data can be found here: [https://dpt2.samhsa.gov/treatment/directory.aspx], accessed on 14 April 2021.

## Appendix A

The approach for the two-step floating catchment area (2SFCA) method is expressed as follows:

**Step 1:** Generate a 30 min drive time zone (catchment) concerning the provider site and compute the provider-to-population ratio at each provider location,

(A1) $$R_{j}=\frac{S_{j}}{\underset{i\in \left\{d_{ij}\leq d_{0}\right\}}{\sum }P_{i}}$$

where:

- $R_{j}$ is the provider-to-population ratio at physician location *j*;
- $P_{i}$ is a population of block group *i*;
- *d*_0_ is a travel threshold; $d_{ij}$ is travel time between i and *j*.

**Step 2:** Generate another 30 min drive time catchment concerning the population site and compute the spatial accessibility index ($A_{i})$ for each population site,

(A2) $$A_{i}=\underset{j\in \left\{d_{ij}\leq d_{0}\right\}}{\sum }R_{j}$$

where:

- $R_{j}$ is the provider-to-population ratio at physician location *j*;
- $R_{j}$ is a travel threshold;
- $d_{ij}$ is travel time between *i* and *j*;
- $A_{i}$ is a spatial accessibility index of each population site *i*.

## Appendix B

**Category**	**Service**
Payment/Insurance/Funding Accepted	Medicare
	Medicaid
	Military insurance (e.g., TRICARE)
	State-financed health insurance plan other than Medicaid
Payment Assistance Available	Payment assistance (check with facility for details)
Ancillary Services	Assistance with obtaining social services
	Residential beds for clients children
	Child care for clients children
	Domestic violence services-family or partner
	Housing services
	Transportation assistance
Detoxification	Benzodiazepines Detoxification
	Cocaine Detoxification
	Opioid Detoxification
	Methamphetamine Detoxification
Counseling Services and Education	Individual counseling offered
	Group counseling offered
	Family counseling offered
	Marital/couples counseling offered
	Substance abuse education
Language Services	Services for the deaf and hard of hearing
	American Indian or Alaska Native languages
	Other languages (excluding Spanish)
	Spanish
Type of Opioid Treatment	SAMHSA-certified Opioid Treatment Program
	Buprenorphine used in Treatment
	Buprenorphine detoxification
	Buprenorphine maintenance
	Buprenorphine maintenance for predetermined time
	Naltrexone used in Treatment
	Methadone detoxification
	Methadone maintenance
	Methadone maintenance for predetermined time
	Prescribes/administers buprenorphine
	Prescribes/administers naltrexone
	Relapse prevention from naltrexone
	Use methadone/buprenorphine for pain management or emergency dosing
	Accepts clients on opioid medication
	Do not use medication for opioid addiction
	Does not treat opioid addiction
